# Serum IGF1 and linear growth in children with congenital leptin deficiency before and after leptin substitution

**DOI:** 10.1038/s41366-021-00809-2

**Published:** 2021-05-17

**Authors:** Marianna Beghini, Stephanie Brandt, Ingrid Körber, Katja Kohlsdorf, Heike Vollbach, Belinda Lennerz, Christian Denzer, Shlomit Shalitin, Ferruccio Santini, Werner F. Blum, Julia von Schnurbein, Martin Wabitsch

**Affiliations:** 1 Division of Pediatric Endocrinology and Diabetes, Department of Pediatrics and Adolescent Medicine, University Medical Centre Ulm, Ulm, Germany; 2 Pediatric Endocrinology and Diabetology Division, Children’s Hospital, University of Bonn, Bonn, Germany; 3 Division of Pediatric Endocrinology, Department of Pediatrics, Boston Children’s Hospital, Boston, MA USA; 4 The Jesse and Sara Lea Shafer Institute of Endocrinology and Diabetes, Schneider Children’s Medical Center of Israel, Petach Tikva, Israel; 5 Sackler Faculty of Medicine, Tel Aviv University, Tel Aviv, Israel; 6 Obesity and Lipodystrophy Center at the Endocrinology Unit, University Hospital of Pisa, Pisa, Italy; 7 Center of Child and Adolescent Medicine, Justus-Liebig University, Giessen, Germany; 8 Present Address: Division of Endocrinology and Metabolism, Department of Medicine III, Medical University of Vienna, Vienna, Austria

**Keywords:** Obesity, Endocrine system and metabolic diseases

## Abstract

**Background:**

Evidence from in vitro and rodent studies suggests that leptin, a key signal of long-term energy reserves, promotes IGF1 synthesis and linear growth. This effect of leptin has not been fully investigated in humans. The aim of our study was to investigate the effect of leptin substitution on growth factors and linear growth in children with congenital leptin deficiency (CLD).

**Methods:**

In this cohort study we included eight pediatric patients (six males), age 0.9–14.8 years, who were diagnosed with CLD and received leptin substitution at our University Medical Center. We calculated standard deviation scores (SDS) for serum levels of IGF1 and IGFBP3, IGF1/IGFBP3 molar ratio, and height at baseline (T0) and 12 months (T12) after the initiation of substitution with metreleptin.

**Results:**

All patients had severe obesity (BMI-SDS mean ± SD: 4.14 ± 1.51) at T0 and significant BMI-SDS reduction to 2.47 ± 1.05 at T12. At T0, all patients were taller than the mid-parental median, yet had low IGF1 and IGF1/IGFBP3 molar ratios (IGF1-SDS$\overline x$_T0_: −1.58 ± 0.92, IGF1/IGFBP3 molar ratio-SDS$\overline x$_T0_: −1.58 ± 0.88). At T12, IGF1-SDS increased significantly (∆_T0–12_: 1.63 ± 1.40, *p* = 0.01), and IGFBP3-SDS and IGF1/IGFBP3 molar ratio-SDS showed a trend toward an increase. In the three children within the childhood growth period (post-infancy, pre-puberty) height-SDS increased (∆height-SDS_T0–12_: 0.57 ± 0.06, *p* = 0.003) despite substantial weight loss.

**Conclusions:**

These results in CLD patients are contrary to observations in children with idiopathic obesity who typically have above-mean IGF1 levels that decrease with weight loss, and therefore suggest that leptin increases IGF1 levels and promotes linear growth.

## Introduction

Linear growth is associated with significant energy expenditure. Therefore, it is not surprising that IGF1, a major regulator of growth, is modulated by both acute and chronic nutritional status [1]. Leptin, a key signal of long-term energy availability that correlates with fat mass, has been implicated in the regulation of IGF1 secretion and linear growth during acute and chronic nutritional alterations [2–4]. However, a direct effect of leptin on IGF1 and growth in humans has not been demonstrated to date.

Up to now, evidence pointing toward a direct effect of leptin on IGF1 and growth is mainly derived from in vitro [5, 6] and rodent studies [7–10]. Human data are limited to observed associations of leptin, IGF1 levels, and growth in several human conditions associated with hyper- [11] or hypoleptinemia [1, 3, 12–16], and their response to leptin treatment. Childhood obesity, a state of hyperleptinemia, is associated with normal [17–19] or high [20, 21] levels of IGF1 and accelerated growth, despite a decrease in spontaneous and stimulated GH secretion [11]. On the flipside, states of acute or chronic hypoleptinemia (e.g., acute caloric restriction [3, 15, 16], malnutrition [1], anorexia nervosa [12], hypothalamic amenorrhea [3, 13], and generalized or severe acquired lipodystrophy [14]) are associated with low IGF1 levels. In an interventional placebo-controlled study in 14 fasted healthy adults, leptin administration blunted the starvation-associated decrease of IGF1 levels [3]. Likewise, leptin therapy in patients with hypothalamic amenorrhea [3, 13] and lipodystrophy [14] leads to increases in IGF1 concentrations. These disease models suggest a stimulatory effect of leptin on IGF1, but are confounded by other metabolic alterations that underlie or accompany the altered levels of leptin.

A unique model to clarify leptin effects on the IGF system and linear growth in humans is represented by patients affected by congenital leptin deficiency (CLD), an extremely rare disease caused by biallelic mutations in the leptin gene leading to defective leptin production [22] or leptin bioinactivity [23]. The primary lack of functional leptin signaling in CLD causes hyperphagia and severe obesity [22], so that CLD is distinguishable from physiologic states of low leptin associated with negative energy balance/starvation.

The aim of the present study was to retrospectively analyze the effects of 12 months of leptin substitution on the IGF system and linear growth in a cohort of eight pediatric patients with CLD. Our hypothesis was that leptin substitution would be associated with an increase in IGF1-, IGFBP3-, IGF1/IGFBP3 molar ratio- and height-standard deviation score (SDS). Concomitant changes in BMI, pubertal development (Tanner stage and hormones of the pituitary-gonadal axis), glucose, C-peptide, insulin, and thyroid hormones during leptin substitution were also evaluated as these factors play a role in the regulation of the IGF system and growth.

## Subjects and methods

### Study population

Our cohort comprised six children (five males) and two adolescents (one male) with CLD. The genetic background was Austrian in four children, German in two children and Pakistani in two children as well. No siblings were included in the cohort. The causes of CLD are biallelic homozygous rare variants in the leptin gene resulting in defective leptin production in five patients (c.313C>T, p.Arg105Trp, ID_1 and ID_2; c.398delG, p.Gly133Valfs*15, ID_5 and ID_6; c.215T>C, p.Leu72Ser; ID_8), and in leptin bioinactivity in three patients (c.298G>T, p.Asp100Tyr, ID_3; c.309C>A, p.Asn103Lys, ID_4) (reference sequence Ensembl:ENSG00000174697 or Ensembl:ENST00000308868). Pathogenicity for the variants has been shown in the following publications: p.Gly133Valfs*15 [22], p.Arg105Trp [24], p.Leu72Ser [25], p.Asp100Tyr [23], and p.Asn103Lys [26]. Patient with ID_7 has a yet unpublished biallelic homozygous rare variant in the leptin gene. This variant is probably damaging according to the annotation tools MutationTaster and PolyPhen2. The functional characterization has been performed in our laboratory and is unpublished.

Patients were followed at our outpatient clinic at the Center for Rare Endocrine Diseases, Division of Pediatric Endocrinology and Diabetes, Department of Pediatrics and Adolescent Medicine, University Medical Center Ulm. Our research group has described five of these patients in previous publications (ID_3 (ref. 23), ID_4 (ref. 26, 27), ID_5 and ID_6 (ref. 27), ID_8 (ref. 25, 27, 28)).

Upon confirmation of the genetic diagnosis, informed parental consent was obtained, and leptin substitution was initiated with recombinant methionyl human leptin (metreleptin; Amryt Pharmaceuticals) at a starting dose of 0.03 mg/kg _lean body weight_/day. The examinations adhered to the principles of the Declaration of Helsinki and were approved by the Ethical Committee of the University of Ulm (ethic approval number 177/08, 373/12 and 188/19).

At each outpatient clinic visit, we evaluated anthropometrics (height, weight) and Tanner stage, and collected blood samples.

Here we present data collected at baseline (T0) and after 12 months (T12) of leptin replacement therapy in the eight pediatric patients with CLD. Due to the retrospective nature of this study, the interval between the baseline and the follow-up examination was not exactly 12 months in all patients (mean ± standard deviation (SD): 11.9 ± 1.9 months, range: 10.3–15.5 months).

### Anthropometrics

Body weight was measured to the nearest 0.1 kg on a calibrated balance beam scale (Seca, Hamburg, Germany), and body height was measured to the nearest 0.1 cm (Stadiometer, Busse Design, Ulm, Germany). BMI was calculated by dividing weight (in kg) by the square of height (in m). We calculated BMI and height-SDS using the least mean-squares method based on age and sex specific German reference standards [29, 30].

To interpret height at T0 within the genetic context, we calculated the difference between the SDS of the mid-parental target height [31] and the SDS of patient’s height at T0. To assess changes in linear growth during leptin substitution (T0–T12), we calculated the difference between the height-SDS at T12 and the height-SDS at T0 for the three children above age 1.5 years at T0 who remained prepubertal at T12 (ID_2–4). In this period of childhood growth between infancy and puberty, linear growth is predominantly determined by the GH-IGF1 axis [32] and not confounded by pubertal development that is also impacted by leptin.

In addition, we calculated height velocity (cm/year) for the 6 months before (–T6) ((height_T0_ – height_–T6_ in cm)/(age_T0_ – age_–T6_ in years)) and the 6 months after (T6) ((height_T6_ – height_T0_)/(age_T6_ – age_T0_ in years)) the initiation of leptin substitution.

### Pubertal development

Pubic hair stage (PH) in girls and boys, breast development (B) in girls, and genital stage (G) in boys were assessed using the Tanner and Marshall rating scales by pediatric endocrinologists.

### Laboratory parameters

Blood samples were obtained between 08:00 h and 09:00 h after an overnight fast and were processed by the Department of Clinical Chemistry, University Medical Center Ulm. IGF1 and IGFBP3 serum concentrations were measured with the Immunodiagnostic System (IDS-iSYS, United Kingdom) automated chemiluminescence immunoassay in two patients (ID_1, ID_7) and with Siemens Immulite automated immunoassay (Germany) in the other patients. In order to have homogenous values, we converted all raw serum concentrations measured with Siemens Immulite in IDS-iSYS values through the formulas described by Bidlingmaier et al. IGF1 [33] and by Friedrich et al. for IGFBP3 [34]. With the IDS-iSYS IGF1 and IGFBP3 values we calculated IGF1/IGFBP3 molar ratio × 100 (ref. 34). We then calculated age- and gender-specific SDS of IGF1, IGFBP3, and IGF1/IGFBP3 molar ratio using IDS-iSYS references [33, 34]. The formulas do not take into account the pubertal status. Plasma levels of TSH, total and free T3, total and free T4 were measured with the ElectroChemiLuminescence ImmunoAssay (ECLIA), Roche. Serum glucose concentrations were measured with the hexokinase/glucose-6-phosphate dehydrogenase method. Insulin, C-peptide, luteinizing hormone (LH), follicle stimulating hormone (FSH), estradiol, and testosterone serum concentrations were determined with ECLIA.

### Statistical analysis

Mean and SD are reported. We used a paired *t*-test to analyze changes in BMI-SDS, height-SDS (only in ID_2–4), IGF1-SDS, IGFBP3-SDS, and IGF1/IGFBP3-SDS during leptin substitution. A two-sided *p* value <0.05 was considered as statistically significant. Statistical analysis was performed using the software R 3.0.3 (March 6, 2014; R Core Team, 2014).

## Results

### Age and weight status

**Fig. 1:**
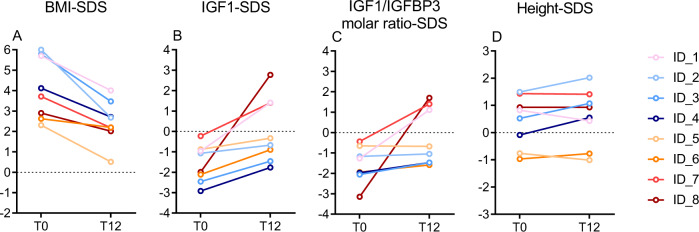
Changes in BMI-, IGF1-, IGF1/IGFBP3 molar ratio-, and height-SDS under leptin substitution. Individual BMI-SDS (**A**), IGF1-SDS (**B**), IGF1/IGFBP3 molar ratio-SDS (**C**), and height-SDS (**D**) at the initiation (T0) and 12 months after (T12) leptin substitution in eight patients with congenital leptin deficiency. SDS standard deviation score.

At the initiation of leptin substitution (T0), age ranged from 0.9 to 14.8 years (Table 1). In the leptin-deficient state (T0), all patients had severe obesity with a mean BMI-SDS_T0_ of 4.14 ± 1.51 (Table 1). After 12 months of leptin substitution, BMI-SDS significantly decreased in all patients ($\overline x$_T12_: 2.47 ± 1.05, *p* = 0.001). Figure 1A illustrates individual changes in BMI-SDS.

**Table 1:** Clinical parameters, IGF1, IGF1-SDS, IGFBP3-SDS, and IGF1/IGFBP3 molar ratio-SDS at the initiation (T0) and 12 months after (T12) leptin substitution in eight patients with congenital leptin deficiency.

	Sex	Age (y)	BMI-SDS			Tanner stage		Height-SDS – mid-parental target height-SDS	Height-SDS			Height velocity (cm/y)^a^		IGF1 (ng/mL)		IGF1-SDS			IGFBP3-SDS			IGF1/IGFBP3 molar ratio-SDS		
		T0	T0	T12	∆T0–12	T0	T12	T0	T0	T12	∆T0–12	−T6	T6	T0	T12	T0	T12	∆T0–12	T0	T12	∆T0–12	T0	T12	∆T0–12
ID_1	M	0.9	5.70	4.01	−1.69	G1, PH1	G1, PH1	1.14	0.82	0.43	−0.39	26.70	12.84	53	155	−0.96	1.41	2.37	1.75	1.19	−0.56	−1.27	1.11	2.38
ID_2	M	1.5	6.00	2.68	−3.32	G1, PH1	G1, PH1	0.86	1.49	2.02	0.53	8.52	17.98	53	73	−1.07	−0.67	0.40	1.02	1.39	0.37	−1.17	−1.05	0.12
ID_3	M	3.0	5.78	3.47	−2.31	G1, PH1	G1, PH1	1.85	0.52	1.07	0.55	8.20	9.80	28	59	−2.46	−1.46	1.00	−0.83	0.19	1.02	−2.06	−1.47	0.59
ID_4	M	6.3	4.12	2.71	−1.41	G1, PH1	G1, PH1	0.09	−0.09	0.55	0.64	8.42	10.35	32	75	−2.92	−1.77	1.15	−2.21	−0.64	1.57	−1.97	−1.47	0.50
ID_5	F	10.8	2.31	0.51	−1.80	B2, PH1	B3, PH3	0.98	−0.76	−1.01	−0.25	n.a.	4.43	163	219	−0.88	−0.33	0.55	−0.44	0.70	1.14	−0.65	−0.68	−0.03
ID_6	M	11.9	2.62	2.20	−0.42	G1, PH1	G3, PH4	2.28	−0.97	−0.77	0.20	n.a.	8.22	117	210	−2.11	−0.90	1.21	−0.05	1.94	1.99	−1.94	−1.59	0.35
ID_7	M	14.7	3.71	2.18	−1.53	G1, PH2	G3, PH5	1.24	1.43	1.41	−0.02	0.00	6.02	295	476	−0.23	1.40	1.63	0.90	0.56	−0.34	−0.44	1.40	1.84
ID_8	F	14.8	2.90	2.01	−0.89	B3, PH5	B5, PH5	1.58	0.93	0.93	0.00	n.a.	1.16	126	677	−1.99	2.77	4.76	1.54	2.19	0.65	−3.15	1.70	4.85
Mean ± SD			4.14 ± 1.51	2.47 ± 1.05	−1.67 ± 0.89			1.25 ± 0.62	0.42 ± 0.94	0.58 ± 1.04	0.57 ± 0.06^b^					−1.58 ± 0.92	0.06 ± 1.61	1.63 ± 1.40	0.21 ± 1.35	0.94 ± 0.93	0.73 ± 0.89	−1.58 ± 0.88	−0.26 ± 1.41	1.32 ± 1.66
*p* value					0.001						0.003^b^							0.01			0.05			0.06

*SDS* standard deviation score, *SD* standard deviation, *G* genital, *PH* pubic hair, *B* breast, *n.a.* data not available.

^a^Height velocity in the 6 months before (−T6) and in the 6 months after (T6) the initiation of leptin replacement therapy.

^b^Mean change ∆T0–12 (±SD) and *p* value of prepubertal children above age 1.5 years at T0 (ID_2–4).

### IGF1-, IGFBP3-, and IGF1/IGFBP3 molar ratio-SDS

In the leptin-deficient state, all patients had low IGF1 levels and IGF1/IGFBP3 molar ratios, as signified by negative values of IGF1-SDS ($\overline x$_T0_: −1.58 ± 0.92, range −2.92 to −0.23) and IGF1/IGFBP3 molar ratio-SDS ($\overline x$_T0_: −1.58 ± 0.88, range −3.15 to −0.44). IGFBP3-SDS was variable, ranging from −2.21 to 1.75 ($\overline x$_T0_: 0.21 ± 1.35) (Table 1).

After 12 months of leptin substitution, IGF1-SDS significantly increased in all patients ($\overline x$_T12_: 0.06 ± 1.61, ∆_T0–12_: 1.63 ± 1.40, *p* = 0.01). Individual changes of IGF1-SDS are shown in Fig. 1B and Table 1. Similar to IGF1-SDS values, there was a trend toward an increase in IGFBP3-SDS (∆_T0–12_: 0.73 ± 0.89, *p* = 0.05) and IGF1/IGFBP3 molar ratio-SDS (∆_T0–122_: 1.32 ± 1.66, *p* = 0.06) (Fig. 1C and Table 1).

### Height and linear growth

At T0, the mean height-SDS ($\overline x$_T0_: 0.42 ± 0.94) was 1.25 ± 0.62 SDS (range 0.09–2.28, Table 1) above the mid-parental target height-SDS. Height-SDS increased significantly with leptin substitution among the three children within the post-infancy, prepubertal childhood growth period (∆_T0–12_: 0.57 ± 0.06, range 0.53–0.64, *p* = 0.003) (Table 1 and Fig. 1D). In these three children, height velocity in the 6 months after initiation of leptin substitution was higher than in the 6 months before (Table 1).

### Pubertal development

ID_1 (0.9 years at T0) was an infant in the growth period of infancy that is largely GH independent. ID_2–4 (age range at T0: 1.5–6.3 years) were prepubertal at T0, and did not show pubertal development during leptin substitution (Table 1). ID_5 (female, 10.8 years at T0) and ID_6 (male, 11.9 years at T0) were classified as B2 and G1 at T0 respectively. After 12 months of leptin substitution, their pubertal stage had progressed to B3 (ID_5) and G3 (ID_6), respectively. ID_7 (male, 14.7 years) and ID_8 (female, 14.8 years) had delayed pubertal development at T0 (Table 1). ID_7 had a gonadal volume of 2.4 ml left and 2.1 ml right, corresponding to Tanner 1 pubertal stage (G1, prepubertal). ID_8 had a breast development corresponding to Tanner 3 pubertal stage (B3). Leptin substitution resulted in the initiation/progression of puberty, with G3 and B5 Tanner stages at T12 in ID_7 and ID_8, respectively (Table 1). Consistent with the clinical observation, plasma concentrations of LH and FSH increased in ID_5–8 from T0 to T12; furthermore, the older patients showed an increment in testosterone levels (ID_6 and ID_7, data not available for ID_8) as well as in estradiol levels (ID_7 and ID_8).

### Parameters of glucose metabolism and thyroid function

Plasma concentrations of fasting glucose were normal in all patients at T0 as well as T12 (Supplementary Table 1). Fasting insulin levels at baseline were high, especially in the oldest children and the adolescents (ID_3–8), and decreased during leptin substitution in seven out of eight patients (Supplementary Table 1). Consistently, C-peptide levels showed an overall significant decrease in the six subjects with available data (∆_T0–12_: −1.4 ± 1.2, *p* = 0.02). Plasma levels of TSH, total and free T3 as well as total and free T4 were in the normal range in all CLD patients at T0, and showed no changes during leptin substitution (Supplementary Table 1).

## Discussion

The circulating growth factors studied in this cohort of children with CLD show impressive changes before and after substitution with leptin. In the untreated state, serum IGF1-SDS and IGF1/IGFBP3 molar ratio-SDS were unexpectedly low although patients were severely obese. Despite a marked weight loss, we documented increases in IGF1-SDS and in IGF1/IGFBP3 molar ratio-SDS during leptin substitution. The fact that IGF1-SDS values increased significantly in our cohort, whereas IGFBP3-SDS values showed a trend toward increase, is consistent with the knowledge that IGF1 usually responds more markedly to stimuli than IGFBP3 [35].

Our findings are in marked contrast to those reported in children with idiopathic, hyperleptinemic obesity who usually have normal [17–19], or increased [20, 21] IGF1 serum levels, whereas weight loss in these children is typically associated with either a decrease [20, 36] or no change in IGF1 levels [37, 38]. Therefore, our results support the hypothesis that leptin as an energy sensor is able to regulate growth factors in humans as shown in animals [7–10]. From a physiological point of view, this function of leptin can be expected. Leptin, which signals starvation when present at very low concentrations, controls mechanisms with high energy demand such as puberty, fertility, and presumably longitudinal growth. Interestingly, leptin substitution resulted in an increase in height-SDS in all three children who were in the childhood growth period that is predominantly determined by GH and IGF1 [32]. In these children we documented also an increase in growth velocity despite pronounced weight loss.

CLD provides an excellent model to investigate the physiological actions of leptin in humans. Considering the rarity of CLD, our cohort with eight children is a relatively large study group providing valuable information. Our study is the first that specifically evaluated IGF1-SDS values in CLD patients allowing a comparison of data independent of age and sex. Raw serum levels of IGF1 (IU/l) of three Pakistani CLD children (child A, B, and C) were described in 2002 by Farooqi et al. [39]. Leptin substitution was initiated approximately at age 9 in child A, and at age 4 in children B and C. Interestingly, in the leptin naive state, the IGF1 serum levels of all three children were close to the lower limit of the normal range. After 36 months of leptin substitution, concomitant with weight loss, IGF1 serum levels rose to the middle of the normal range in child B and reached the upper limit in child A [39], suggesting a greater than the expected age-related increase. These observations are consistent with our data and further support the hypothesis that leptin stimulates IGF1 secretion in patients with CLD.

The increased leptin naive height-SDS in our patients is in line to observations in children with simple obesity [11]. Therefore, we speculate that the accelerated growth in the children with CLD at T0 may be linked with their obesity by leptin-independent mechanisms, such as hyperinsulinism. Further data supporting a leptin-independent effect of weight excess on skeletal maturation and growth in CLD derive from the four children in Farooqi’s cohort who all showed an advanced bone age in the untreated state [39, 40]. Without information regarding height-SDS and mid-parental target height-SDS, we cannot be sure that linear growth was accelerated; however, the advanced bone age hints at this possibility. The advanced bone age in the four children [39, 40] and the fact that growth was not impaired in our eight patients suggest that leptin is not necessary for longitudinal growth per se, at least not if obesity is present. Accordingly, Licinio et al. [41] reported normal height commensurate with that of unaffected relatives in three untreated adults with CLD.

Interestingly, leptin substitution resulted in an increase in height-SDS in all three children who were in the childhood growth period between infancy and puberty, indicating an increment in height velocity. In line with our findings, Licinio’s research group [42] previously presented a boy with CLD who lost weight during leptin treatment and simultaneously showed an increase in height from the 10th to the 25th percentile. Previous studies found that children with idiopathic, hyperleptinemic obesity showed a height deceleration during weight loss [43, 44]. During negative energy balance, leptin also decreases markedly [45]. These findings support the idea that the increase in leptin levels could explain the height acceleration concomitant with weight loss in the CLD patients.

In addition to being a key factor in the regulation of energy homeostasis, leptin plays a permissive role in regulating pubertal development and fertility. Consistently, pubertal development in the two young adolescents of our cohort (ID_7 and ID_8) was delayed in the leptin-deficient state, and progressed once leptin substitution was initiated (details regarding the effect of leptin substitution on pubertal development in ID_8 have been described [28]). Two patients of our cohort (ID_5 and ID_6) progressed through normally timed puberty during leptin substitution. Since the SDS values allowed us to adjust only for age and gender but not for pubertal stage, it is possible that the delayed puberty in ID_7 and ID_8 may explain the low IGF1-SDS; moreover, for these two adolescents and the two peripubertal children we cannot rule out that the pubertal development contributed to the increase in IGF1-SDS values. Nevertheless, since we observed a rise in IGF1-SDS in the other four children as well, we believe that the increment in IGF1-SDS is not entirely attributable to the effects of leptin on puberty.

Fasting insulin levels at baseline were increased, and declined in seven out of eight patients during leptin substitution most likely reflecting a reduction in obesity-associated insulin resistance. Insulin can act on the IGF1-receptor [46], and hyperinsulinemia is thought to be a factor stimulating growth in idiopathic childhood obesity [47] and in hypothalamic obesity in which GH is missing [48]. In this setting, high levels of insulin could explain the height-SDS higher than expected in our patients in the untreated state. Consistently, tall stature has been described in patients with lipodystrophy who exhibit low levels of leptin and high levels of insulin [49, 50].

However, in contrast to the increase in growth velocity we observed in our three children within the childhood growth period, Brown et al. found a decrease in growth velocity after 1 year of metreleptin treatment in children with lipodystrophy [50]. The authors proposed that the reduction in height velocity under leptin treatment may be due to the drop in insulin levels that follows the improvement in insulin resistance [50]. We speculate that the clear increase in IGF1 plasma concentrations in our patients compensated for the reduction in insulin levels, resulting in a net increment in the stimulation of IGF1 receptors. Moreover, glucose metabolism was markedly more compromised in the pediatric patients with lipodystrophy (diabetes was reported in 32% in children <12 years, 94% in adolescents between 12 and 18 years) [50] than in our study subjects who all had normal fasting glucose plasma levels in the untreated state. Therefore, it is possible that the impact of insulin changes may have been more prominent in the lipodystrophic cohort than in ours.

We found no changes in levels of TSH, total and free T4 and T3 during leptin substitution in our cohort of CLD patients. Therefore, it is unlikely that thyroid hormones played a role in stimulating IGF1 synthesis.

There are some meaningful experimental findings that may explain the growth stimulating role of leptin. While IGF1 can be produced by most organs, the liver is the primary source of circulating IGF1 [35]. In vitro studies have shown that leptin stimulates IGF1 synthesis in chondrocytes [5, 51], which is thought to have mostly paracrine function. The increased systemic IGF1 levels with leptin substitution in our patients however unlikely derive from local bone action alone, and suggest an effect of leptin on liver IGF1 secretion. In the following paragraphs we discuss three potential mechanisms by which leptin may stimulate hepatic IGF1 synthesis: (i) stimulation of pituitary GH secretion, (ii) direct action on hepatic leptin receptors, or (iii) sensitization of liver GH receptors.

GH secreting neurons express leptin receptors, and leptin stimulates both spontaneous pulsatile GH secretion and the GH response to GHRH in rodents [52], However, this stimulatory effect has not been replicated in humans [3, 53]. Reduced GH secretion was previously described in three untreated CLD patients in the setting of normal growth [41]. Similarly, in a previous case report on patient ID_8 [28], we have described that both spontaneous and stimulated GH secretion were decreased in the untreated state and normalized with leptin substitution. However, in line with Ozata et al. [41], it could also be suggested that the state of obesity per se, rather than the lack of leptin, was responsible for the reduced GH secretion, and the normalization in the GH secretion resulted from the weight loss under leptin substitution [28]. A GH-independent effect of leptin on IGF1 secretion is also suggested by the finding that leptin administration blunted starvation-associated decreases of IGF1 without affecting GH levels after a 72-h fast in healthy adults [3].

Human hepatocytes express the long form of leptin receptor [54], such that direct stimulation of hepatocytes to secrete IGF1 is a possibility. In addition, leptin may increase hepatic GH-sensitivity, as suggested by the GH-resistance seen in conditions associated with acute [3, 15, 16] and chronic hypoleptinemia [1, 12], and the increased GH responsiveness associated with idiopathic, hyperleptinemic obesity [55, 56]. Hepatic GH-sensitivity may be influenced by leptin in different ways including via modulation of autonomic tone, via an increase in insulin sensitivity [57, 58], or directly by stimulating leptin receptors.

It has to be noted that our study is focused on IGF1 as the main growth factor. Since linear growth is a complexly regulated process and an interplay of other endocrine and paracrine factors is involved, leptin may also contribute to linear growth regulation via other mechanisms, including local proliferative effects on epiphysial growth plates, as suggested by several in vitro and rodent experiments [5, 6, 51, 59].

A limitation of our study is the absence of a weight-matched control group. Nevertheless, our results were, based on the available literature, contrary to expectations for children with idiopathic obesity and are therefore unlikely to be explained by obesity per se. Due to the retrospective nature of the study, GH nocturnal secretion profile was available only for one patient, not allowing to further explore GH-IGF1-interactions.

Taken together, our results support the hypothesis that leptin, as a key signal of energy availability, promotes IGF1 secretion in relation to the nutritional status. Our results in patients with CLD are contrary to findings in children with idiopathic, hyperleptinemic obesity who typically have normal or high IGF1 that decreases with weight loss, and suggest that in patients with CLD leptin increases IGF1 levels and promotes linear growth. Further studies are needed to confirm our findings and to elucidate the exact underlying mechanisms by which leptin stimulates IGF1 secretion and growth.

## Supplementary information

Supplementary Table 1
